# Inactivation of XPF Sensitizes Cancer Cells to Gemcitabine

**DOI:** 10.1155/2019/6357609

**Published:** 2019-03-03

**Authors:** Joseph W. George, Mika Bessho, Tadayoshi Bessho

**Affiliations:** The Eppley Institute for Research in Cancer and Allied Diseases, Fred & Pamela Buffett Cancer Center, University of Nebraska Medical Center, 986805 Nebraska Medical Center, Omaha, NE 68198-6805, USA

## Abstract

Gemcitabine (2′, 2′-difluorodeoxycytidine; dFdC) is a deoxycytidine analog and is used primarily against pancreatic cancer. The cytotoxicity of gemcitabine is due to the inhibition of DNA replication. However, a mechanism of removal of the incorporated dFdC is largely unknown. In this report, we discovered that nucleotide excision repair protein XPF-ERCC1 participates in the repair of gemcitabine-induced DNA damage and inactivation of XPF sensitizes cells to gemcitabine. Further analysis identified that XPF-ERCC1 functions together with apurinic/apyrimidinic endonuclease (APE) in the repair of gemcitabine-induced DNA damage. Our results demonstrate the importance of the evaluation of DNA repair activities in gemcitabine treatment.

## 1. Introduction

Gemcitabine (2′, 2′-difluorodeoxycytidine; dFdC) is a deoxycytidine analog and frequently used against various solid tumors. Particularly, gemcitabine is a very important chemotherapeutic for the treatment of pancreatic cancer because there are very few options available for this deadly cancer [1]. Gemcitabine can be used both alone (monotherapy) and in combination with other treatments such as gamma-ray irradiation and platinum compounds [2]. As is the case for other cancer chemotherapies, outcomes of gemcitabine treatment vary among patients due to intrinsic and acquired resistance to gemcitabine [3]. Thus, identification of genetic factors that influence the efficacy of gemcitabine is desired.

Metabolism and mechanisms of action of gemcitabine have been studied to some extent [1, 3]. It is believed that the cytotoxicity of gemcitabine is due to the inhibition of DNA replication. Because gemcitabine is a nucleoside analog, it requires active and specialized transportation into cells. Then, gemcitabine is phosphorylated to dFdCTP that can be incorporated by DNA polymerases and inhibits DNA synthesis. Any factors that modulate these steps could determine the efficacy of gemcitabine [1, 3]. Correlations of expression and/or activity levels of concentrative nucleoside transporters (CNTs), equilibrative nucleoside transporters (ENTs), deoxycytidine kinase (dCK), thymidine kinase 2 (TK2), and deoxycytidine deaminase (dCDA) to cytotoxicity of gemcitabine have been reported. These data confirm that efficiencies of the transportation of gemcitabine into cells and activities to phosphorylate gemcitabine to dFdCTP are the determinant of efficacy of gemcitabine treatment.

The early biochemical studies demonstrated a unique feature of the gemcitabine-induced inhibition of DNA synthesis [4]. Unlike other chain-terminating nucleoside analogs (CTNAs), such as araC (cytarabine), ACV (acyclovir), and ddC (zalcitabine), which block an incorporation of a next incoming dNTP, DNA polymerases can incorporate a single deoxynucleotide from the primer end with dFdCMP and then the synthesis is blocked afterwards (masked chain-termination). Once incorporated by DNA polymerases during replication, dFdCMP blocks chain elongation. Because dFdCMP is resistant to the exonucleolytic proofreading activity of DNA polymerases [4], dFdCMP is left near the 3′-end of the primer. Thus, a major gemcitabine-induced DNA damage will be a single strand break (SSB) with a dFdCMP at or near the 3′-end. Interestingly, it was also demonstrated that the “masked chain-termination” might be sequence context-dependent and DNA synthesis could proceed without inhibition [5, 6]. These data implicate that dFdCMP can be incorporated into the genome and served as a template for the next round of DNA replication. Importantly, dFdCMP in the template also blocks DNA synthesis by DNA polymerases in vitro [7]. Therefore, gemcitabine exerts the cytotoxic action by two different mechanisms, inhibition of the extension of a primer and blocking DNA replication in the template strand. How dFdCMP near a terminated primer end or in a template strand is removed is ill defined.

The XPF-ERCC1 complex is a structure-specific endonuclease and plays multiple roles in various DNA repair pathways [8–10]. The complex is responsible for the 5′ incision to a DNA lesion during nucleotide excision repair, releases a cross-linked strand from DNA interstrand crosslinks (ICLs) during ICL repair [11–15], and is essential for single strand annealing (SSA) [16–18]. The XPF-ERCC1 complex is also involved in single strand break (SSB) repair induced by reactive oxygen species (ROS) [19]. Because one of the gemcitabine-induced DNA lesions could be SSBs with dFdCMP near a terminated primer end, we investigated the impact of XPF on the cytotoxicity of gemcitabine. XPF-deficient cells are sensitive to gemcitabine. A genetic epistasis study demonstrated that XPF functions in the same pathway as AP endonuclease (APE) that is a major player in SSB repair. Furthermore, we discovered that the recruitment of XPF to chromatin after the gemcitabine treatment depends on APE. These results showed that XPF and APE are in the same DNA repair pathway for gemcitabine-induced DNA damage and XPF might be required to process an intermediate DNA structure generated by APE.

## 2. Materials and Methods

### 2.1. Cell Lines

CHO UV41, UV135, HeLa S3, and BxPC3 were purchased from American Type Culture Collection (ATCC). HCT116 and HCT116 shAPE (expresses APE1 shRNA constitutively to suppress APE expression) were generous gift from Dr. Kishor Bhakat (University of Nebraska Medical Center). BRCA2-deficient ovarian cancer cell line PE01 and its BRCA2 revertant PE01(C4-2) and BRCA2-deficient pancreatic cancer cell line CAPAN1 and its BRCA2 revertant CAPAN1(C2-1) were generous gift from Dr. Toshiyasu Taniguchi at Tokai University (Japan).

HeLa, BxPC3, PE01, PE01(C4-2), CAPAN1, and CAPAN1(C2-1) were grown and maintained in DMEM high glucose (Hyclone) supplemented with 10% Fetal Bovine Serum (FBS, Invitrogen). HCT116 and its derivative cell lines were grown in McCoy's 5A (Hyclone) supplemented with 10% FBS. All cell lines were cultured at 37°C in 5% humidified CO2 incubators.

### 2.2. Cellular Sensitivity to DNA Damaging Agents – Clonogenic Survival Assay

Cells were seeded in 12-well plates at 300~500 cells/well. After growing one day, gemcitabine was added at the indicated concentrations. The cells were grown 5~7 days in the presence of gemcitabine. For UV treatment, after growing one day, medium was removed from each well and cells were washed with 1 ml of PBS twice. After removing PBS, cells were irradiated by UVC (254 nm) at the dose indicated. Fresh medium was added immediately to each well after the irradiation and incubated an additional 5~7 days. For mitomycin C (MMC) treatment, after growing one day, MMC was added to the indicated concentrations and the cells were incubated for two hours. After washing the cells with PBS twice, fresh medium was added to each well and incubated for 5~7 days. For olaparib treatment, olaparib was added at the indicated concentrations and the cells were grown 5~7 days in the presence of olaparib. Colonies were fixed with ethanol, stained with a Crystal violet solution, and counted. The surviving fractions were calculated by dividing the number of colonies on treated wells by the number on untreated cells. Each surviving fraction with standard deviation from each experiment is listed in Supplementary Table 1.

### 2.3. siRNA Treatment

For the suppression of XPF and APE, siXPF (Dharmacon siGENOME ERCC4 siRNA D-019946-04) and siAPE (Dharmacon ON-TARGET PLUS siRNA HUMAN APEX1 J-010237-07) were used. Cells were seeded at 10^5^ cells per well in a six-well plate one day before siRNA treatment. The cells were treated with 100 nM siRNA for 5 hr. DharmaFECT1 (Dharmacon) was used for the transfection. For the cosuppression of XPF and APE, 100 nM of each siRNA was mixed. After removing the siRNA, the cells were grown for 48 hours in fresh medium. These siRNA-treated cells were used for clonogenic survival assay. A nontargeting control siRNA was used as control.

### 2.4. Generation of XPF-Deficient Mutants by CRISPR/Cas9

XPF-deficient HCT116 cells were generated by CRISPR/Cas9 technology.

Guide sequence for XPF (5′-gccggctcgacggattgcca-3′) was cloned into the transfer plasmid, pLentiCRISPR v2 (from GenScript). Lentiviral particles were generated by using ViralPower Lentiviral expression systems with 293FT cells (Invitrogen). The lentivirus was infected into HCT116 and HCT116 shAPE. Single colonies were established in the presence of puromycin (2 *μ*g/ml) and the expression of XPF was monitored by western blots (Supplementary Figure 3C). XPF-inactivated clones displayed UV sensitivity due to a NER defect (Supplementary Figure 3A).

### 2.5. Preparation of Chromatin Fraction

Cells were harvested after the indicated incubation time with or without the treatment with gemcitabine and resuspended into 2 x cell pellet volume (CPV) of buffer A (10 mM Tris-HCl pH 7.9, 0.34 M sucrose, 3 mM CaCl_2_, 2 mM Mg-acetate, 0.1 mM EDTA, 0.5% NP-40, 1 mM DTT, and protease inhibitors). After incubation for 30 min with rocking at 4°C, nuclei were collected in pellet 1 (P1) by low-speed centrifugation at 3,500 x g for 15 min at 4°C. The supernatant (S1: cytoplasmic lysate) was clarified by high-speed centrifugation at 20,000 × g for 15 min at 4°C. After washing the P1 with 500 *μ*l of buffer A without NP-40, P1 was resuspended into 2 x CPV of buffer B (20 mM HEPES-KOH pH7.9, 3 mM EDTA, 10% glycerol, 150 mM potassium-acetate, 1.5 mM MgCl_2_, 0.1% NP-40, 1 mM DTT, and protease inhibitors). Insoluble chromatin pellet (P2) was prepared by homogenizing nuclei by passing 15 strokes through a 26-gauge syringe needle on ice followed by high-speed centrifugation at 15,000 x g for 30 min at 4°C. The supernatant (S2: nuclear lysate) was clarified by high-speed centrifugation at 20,000 × g for 15 min at 4°C. P2 was resuspended into 100 *μ*l of 0.2 M HCl and incubated for 10 min on ice. After neutralizing pH by adding 20 *μ*l of 1.5 M Tris-HCl (pH 8.8), the supernatant was clarified by high-speed centrifugation at 20,000 × g for 15 min at 4°C and used as chromatin fraction (S3) (Supplementary Figure 4).

### 2.6. Western Blots

Whole cell lysates were prepared for western blots. Harvested cells were resuspended into 2 x CPV (cell pellet volume) of cell lysis buffer (PBS with 1 % NP-40, 1 % Triton X100, and 10% glycerol) and incubated for 15 min at 4 °C. After centrifugation at 16,000 g for 10 min at 4°C, the supernatant was used as whole cell lysate.

Cell lysates (10-100 *μ*g) were separated in SDS-gels and proteins were transferred to membranes. After blocking with 5% nonfat dry milk in TBST, the membranes were incubated with the indicated primary antibodies. The signals were obtained with ECL (Bio-Rad) on X-ray films. Primary antibodies used are anti-XPF antibody (XPF Ab-1 clone 219 from Thermo Scientific), anti-APE antibody (NB100-116 from Novus Biologicals), anti-H2AX antibody (A300-083A from Bethyl Laboratories), anti-Tubulin (GT114, GeneTex), anti-GAPDH (GT239, GeneTex), and anti-Lamin B (PA5-32474, Thermo Fisher Scientific).

### 2.7. Statistical Analysis

Three independent experiments were performed and the results were shown as the average values and standard deviations. Statistical significance was determined by an unpaired two-tailed Student's* t*-test. A* P*-value<0.05 was considered statistically significant.

## 3. Results

### 3.1. XPF-Deficient Cells are Sensitive to Gemcitabine

**Figure 1:**
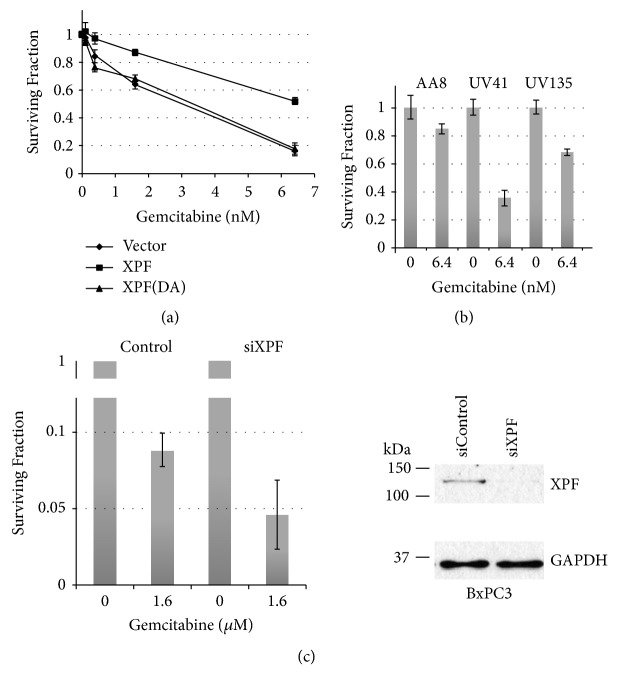
*XPF is required for the cellular resistance to gemcitabine*. Impact of XPF on gemcitabine sensitivity was examined by clonogenic survival assay. Cells were seeded one day before the gemcitabine treatment in 12-well plates. The indicated concentrations of gemcitabine were added and the cells were exposed to gemcitabine throughout the 5~7 days of incubation. After fixing, the cells were stained with Crystal violet. Surviving fraction was calculated by dividing number of cells with gemcitabine by number of cells without gemcitabine. Three independent experiments were performed and averages of surviving fraction are plotted. The error bars show standard deviations.* (a) XPF-deficient UV41 cells are sensitive to gemcitabine and the endonuclease activity of XPF is required for the cellular resistance to gemcitabine*. UV41 with vector alone (closed diamond) are sensitive to gemcitabine compared to the UV41 reconstituted with human XPF gene (closed square). Reconstituted UV41 with human XPF gene (closed square) restored resistance to gemcitabine while the endonuclease-deficient XPF (XPF-DA) (closed triangle) failed to restore the gemcitabine resistance. The difference in the gemcitabine sensitivity at each concentration is statistically significant with P<0.01.* (b) Nucleotide excision repair (NER) contributes to the cellular resistance to gemcitabine*. A NER deficient UV135 showed a moderate sensitivity to gemcitabine compared to the sensitivity in UV41. The differences in the gemcitabine sensitivity at 6.4 *μ*M between AA8 and UV41, AA8 and UV135, and UV41 and UV135 are statistically significant with P<0.05*. (c) Suppression of XPF in pancreatic cancer cell line BxPC3 sensitizes cells to gemcitabine*. The expression of XPF was suppressed by siRNA in pancreatic cancer cell line BxPC3 and the cellular sensitivity to gemcitabine was examined. The difference in the gemcitabine sensitivity between siControl- and siXPF-treated cells is statistically significant with P<0.05. The western blots showed the suppression of XPF. The expression of XPF was significantly reduced (more than 95%) with the siRNA treatment. GADPH was used as a protein loading control.

**Figure 2:**
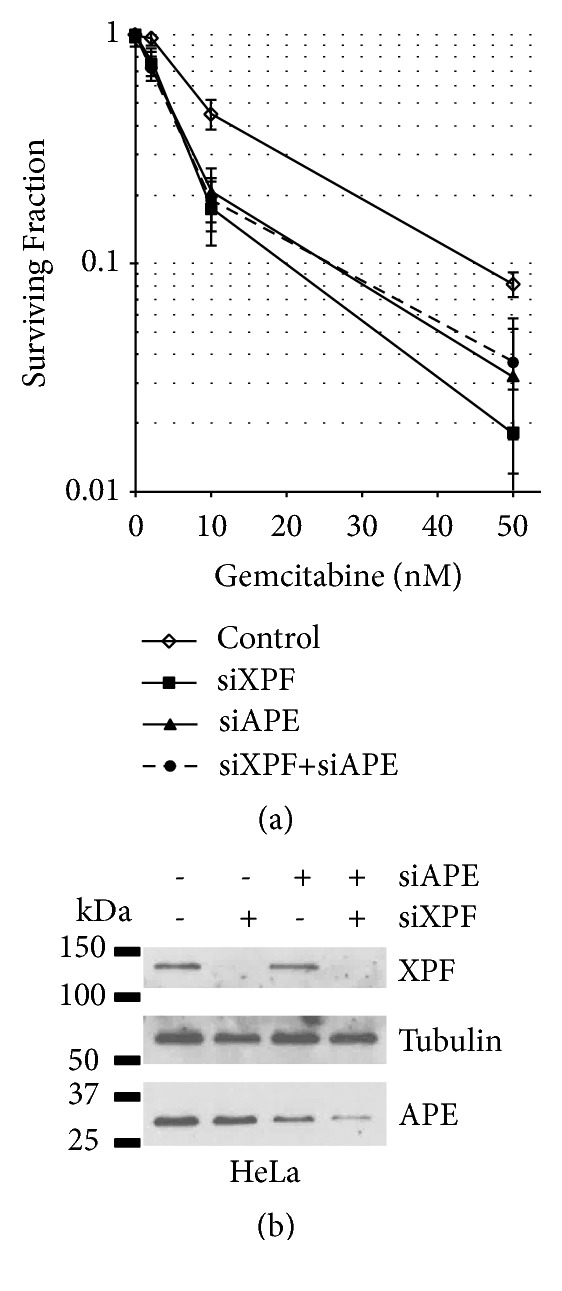
*XPF is epistatic to APE in the gemcitabine resistance*. The expression of XPF and/or APE was suppressed by siRNAs in HeLa cells and the cellular sensitivity to gemcitabine was examined. XPF- or APE-suppressed HeLa cells (closed square and closed triangle, respectively) showed sensitivity to gemcitabine. Cosuppression of XPF and APE (closed circle with dashed line) resulted in the sensitivity to gemcitabine similar to the sensitivity induced by the suppression of XPF or APE individually. A control siRNA (siControl) was used as a control (open diamond). Three independent experiments were performed and averages of surviving fraction are plotted. The error bars show standard deviations. The differences in the gemcitabine sensitivity between the control cells and the cells treated with siXPF, siAPE, or siXPF+siAPE are statistically significant at 10 nM and 50 nM with p<0.05. The western blots showed a significant suppression of XPF (more than 95%) and ~75% reduction in the expression of APE with the siRNA treatment. The cosuppression experiments with two siRNAs, siXPF and siAPE, resulted in similar levels of suppression of each protein induced by individual siRNA (more than 95% reduction in XPF and ~85% reduction in APE). Tubulin was used as a protein loading control.

DNA repair deficient Chinese hamster ovary (CHO) cell lines have been used to study the mechanisms of DNA repair. UV41 and UV135 are UV sensitive mutants that are defective in nucleotide excision repair (NER). XPF that makes a 5′-incision to DNA damage during NER is inactivated in UV41, and XPG that makes a 3′-incision to DNA damage is inactivated in UV135. We examined cellular sensitivity to gemcitabine in UV41. We, along with other groups, previously demonstrated that XPF (as a complex with ERCC1) removes 3′-blocking ends and participates in repair of oxidative DNA damage and Camptothecin-induced DNA damage [19–21]. This activity is unique to XPF (and ERCC1) and not shared with other NER factors. The “masked chain-termination” leaves a dFdCMP one nucleotide next to the primer end and the exonuclease activity of DNA polymerases is not able to remove dFdCMP from the primer end. As a result, the 3′-end of primer is blocked by dFdCMP. Thus, XPF is a good candidate to remove the dFdCMP-blocked 3′-end. As shown in Figure 1(a), UV41 is sensitive to gemcitabine and the expression of the wild type human XPF gene in UV41 (Supplementary Figure 1) [12] restored the resistance to gemcitabine (surviving fraction at 6.4 nM gemcitabine: 0.163 ± 0.036 for UV41 with vector and 0.52 ± 0.021 for UV41 with XPF). Furthermore, the expression of the endo-/exonuclease-inactivated XPF (XPF-DA) in UV41 [12] failed to rescue the phenotype (Figure 1(a); surviving fraction at 6.4 nM gemcitabine: 0.52 ± 0.021 for UV41 with XPF and 0.179 ± 0.041 for UV41 with XPF(DA), p<0.01). These data strongly implicate that XPF is involved in the processing of gemcitabine-induced DNA damage. The suppression of the XPF gene in BxPC3 (pancreatic cancer cell line, Figure 1(c), surviving fraction at 1.6 *μ*M gemcitabine: 0.089 ± 0.01 for BxPC3 and 0.041 ± 0.023 for the XPF-suppressed BxPC3, p<0.05), HeLa (cervical cancer cell line, Figure 2, surviving fraction at 50 nM gemcitabine: 0.081±0.01 for HeLa and 0.018±0.02 for the XPF-suppressed HeLa, p<0.01), and HCT116 (colon cancer cell line, Supplementary Figure 3B, surviving fraction at 125 nM gemcitabine: 0.631 ± 0.022 for HCT116 and 0.109 ± 0.043 for XPF-inactivated HCT116 g4-10, <0.01) also sensitized the cells to gemcitabine, confirming the role of XPF in the repair of gemcitabine-induced DNA damage.

XPF is one of the NER factors. Because dFdCMP can be incorporated into duplex DNA, NER might be responsible for removing this lesion. Interestingly, XPG-deficient UV135 also showed a moderate sensitivity to gemcitabine (Figure 1(b), surviving fraction at 6.4 nM gemcitabine: 0.848 ± 0.038 for AA8 and 0.681 ± 0.025 for UV135, p<0.05). The results suggest that NER contributes to repair of gemcitabine-induced DNA damage.

### 3.2. XPF and APE Function in the Same DNA Repair Pathway to Repair Gemcitabine-Induced DNA Damage

Inactivation of XPF confers cells to cellular sensitivity to ROS, strongly implicating the role of XPF-ERCC1 in SSB [19]. APE is one of the major DNA repair factors in SSB repair [22]. The suppression of APE by siRNA in HeLa cells (Figure 2, surviving fraction at 50 nM gemcitabine: 0.081 ± 0.01 for HeLa with siControl and 0.032 ± 0.011 for HeLa with siAPE, p<0.05) confirmed the previous report that APE is also involved in the repair of gemcitabine-induced DNA damage [23]. To examine the genetic relationship between XPF and APE, both XPF and APE were suppressed by siRNA in HeLa cells and the cellular sensitivity to gemcitabine was studied. Suppression of XPF resulted in mitomycin C (MMC) sensitivity (Supplementary Figure 2A). Cosuppression of XPF and APE in HeLa cells resulted in a similar cellular sensitivity to gemcitabine to the sensitivity with the suppression of each single gene (Figure 2, Supplementary Figure 2B). We also obtained similar results using a mismatch repair- (MMR-) deficient colon cancer cell line, HCT116 (Supplementary Figure 3B). HCT116 constitutively suppressed APE by shRNA was used to generate a XPF mutant by CRISPR/Cas9 (Supplementary Figure 3C). The XPF inactivation in APE-suppressed HCT116 did not alter the cellular sensitivity to gemcitabine. We conclude that XPF and APE are genetically epistatic in the repair of gemcitabine-induced DNA damage.

### 3.3. Recruitment of XPF to Chromatin after Gemcitabine Treatment is Dependent on APE

**Figure 3:**
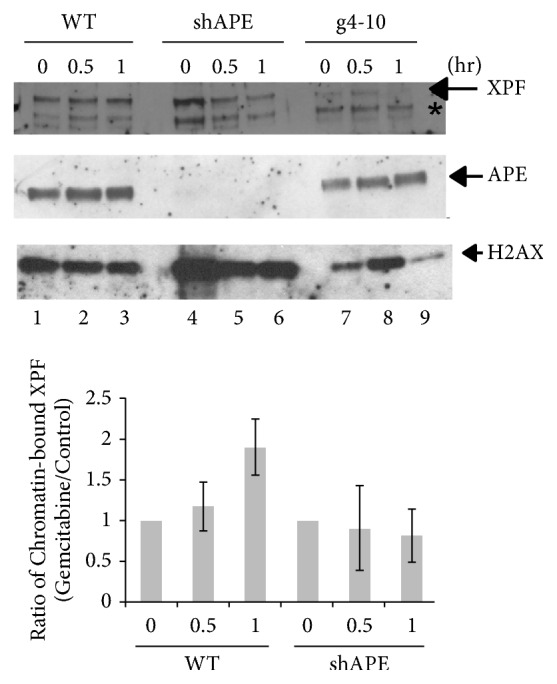
*Gemcitabine-induced recruitment of XPF to chromatin depends on APE*. HCT116, APE-suppressed HCT116 (HCT116 shAPE), and XPF-deficient HCT116 (HCT116 g4-10) cells were treated with 1 *μ*M of gemcitabine and chromatin fractions were isolated in the indicated time. The presence of XPF and APE was detected by western blots. The asterisk (*∗*) shows a cross-reacted protein with the anti-XPF antibody. Histone H2AX was used as a loading control. Gemcitabine-induced recruitment of XPF to chromatin (lanes 1-3) was compromised in HCT116 shAPE cell line (lanes 4-5). The chromatin-bound APE is not changed by the gemcitabine treatment (lanes 4-6, Supplementary Figure 5). A signal of XPF (and APE) was normalized with a signal of H2AX in each chromatin fraction using ImageJ software. Then a ratio of chromatin-bound XPF with gemcitabine to XPF in control was determined and depicted as bar graphs. Three independent experiments were performed and averages of the ratio at indicated time points were plotted. The error bars show standard deviations. Only the difference in the chromatin-bound XPF between chromatin from control experiments and chromatin that was incubated one hour with 1 *μ*M gemcitabine is statistically significant in HCT116 (p<0.05).

To understand the mechanism of the repair of gemcitabine-induced DNA damage, we investigated the recruitment of XPF to chromatin after gemcitabine treatment. The gemcitabine treatment induced an accumulation of XPF on chromatin in HCT116, while gemcitabine did not change the amount of chromatin-bound APE (Figure 3, Supplementary Figure 5). This recruitment of XPF was significantly diminished in APE-suppressed HCT116 (Figure 3). The data strongly indicate that XPF was recruited to a DNA repair intermediate generated after the action of APE.

### 3.4. BRCA2 Is Required for the Cytotoxicity of Gemcitabine

**Figure 4:**
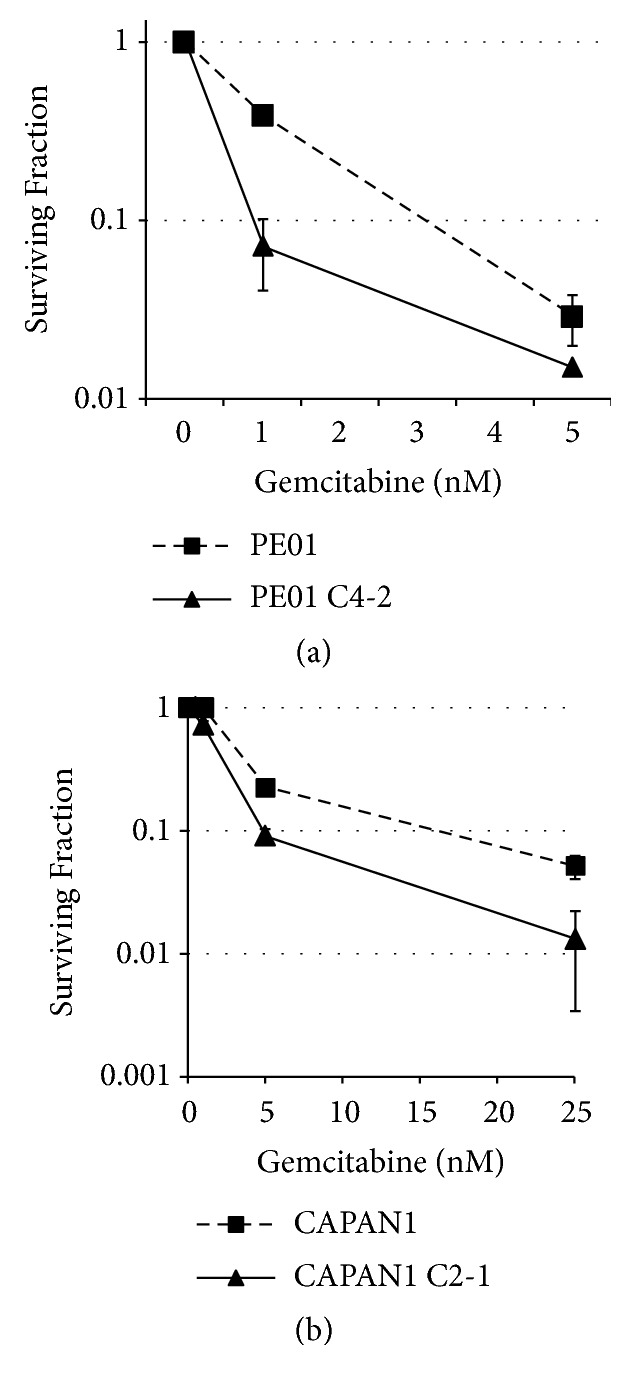
*BRCA2 mediates the cytotoxicity induced by gemcitabine*. Impact of BRCA2 on the cellular sensitivity to gemcitabine was examined. BRCA2-deficient ovarian cancer cell line PE01 (closed square with dashed line) and pancreatic cancer cell line CAPAN1 (closed square with dashed line) are more resistant to gemcitabine compared to PE01(C4-2) (closed triangle) and CAPAN1 (C2-1) (closed triangle) that regain active BRCA2, respectively. Three independent experiments were performed and averages of surviving fraction are plotted. The error bars show standard deviations. The differences in the gemcitabine sensitivity between PE01 and PE01(C4-2), or CAPAN1 and CAPAN1(C2-1), are statistically significant with p<0.05.

Unrepaired SSBs are converted to double strand breaks (DSBs) after a next round of DNA replication. Thus, a defect in homologous recombination (HR) is expected to sensitize cells to gemcitabine. However, previous reports indicated that homologous recombination (HR) is indeed required for the cytotoxicity of gemcitabine [24, 25]. We examined sensitivity to gemcitabine in BRCA2-defective ovarian cancer cell line PE01 and its BRCA2-revertant PE01(C4-2). PE01(C4-2) is one of the PARP inhibitor-cross-resistant clones obtained by the long exposure of BRCA2-mutated PE01 to cisplatin. BRCA2 is restored and HR is fully active in PE01(C4-2) [26] (Supplementary Figures 6A and 6B). Our data demonstrated that BRCA2-deficient PE01 is more resistant to gemcitabine, compared to the BRCA2-restored, HR active PE01(C4-2) (Figure 4(a), surviving fraction at 1 nM gemcitabine: 0.385±0.016 for PE01 and 0.071±0.031 for PE01(C4-2), p<0.01, and at 5 nM gemcitabine: 0.029 ± 0.009 for PE01 and 0.015 ± 0.001 for PE01(C4-2), P<0.05). We also obtained similar results using the BRCA2-deficient pancreatic cancer cell line CAPAN1 and its BRCA2-revertant CAPAN1(C2-1) (Figure 4(b), surviving fraction at 5 nM gemcitabine: 0.229±0.011 for CAPAN1 and 0.091±0.011 for CAPAN1(C2-1), p<0.05, and at 25 nM gemcitabine: 0.051 ± 0.011 for CAPAN1 and 0.013 ± 0.009 for CAPAN1(C2-1), p<0.05). CAPAN1(C2-1) was isolated as one of BRCA2-revertants by selecting cisplatin-resistant CAPAN1 after a long exposure to cisplatin. It was confirmed that the BRCA2 gene is reverted and the HR activity is restored [27] (Supplementary Figure 6C). These data demonstrated clearly that BRCA2 (thus HR) is required for gemcitabine-induced cell death.

## 4. Discussion

The efficacy of gemcitabine varies from patient to patient. Emergence of acquired drug resistance is also a critical issue for gemcitabine treatment. The intrinsic and acquired gemcitabine resistance is caused by various factors including the expression levels of metabolic genes for gemcitabine, tumor microenvironment, and the status of DNA repair activities for gemcitabine-induced DNA damage. Therefore, understanding the mechanisms of each factor/pathway, how they contribute to the cytotoxicity of gemcitabine, and how different factors/pathways cross-talk to enhance or reduce the efficacy of gemcitabine is necessary to develop a more effective regimen. In this report, we found that inactivation of XPF or APE sensitizes cells to gemcitabine and the two DNA repair factors function in the same pathway to remove gemcitabine-induced DNA damage. Interestingly, the gemcitabine-induced recruitment of XPF depends on APE, but the amount of APE on the chromatin is not changed in the absence of XPF. These data implicate that XPF-ERCC1 functions downstream of APE, and XPF is probably recruited to a DNA repair intermediate generated after the action of APE. We did not detect gemcitabine-induced recruitment of APE under the conditions used. Recruitment of APE by gemcitabine might be masked by the APE that is bound to chromatin without DNA damage.

Gemcitabine could inhibit DNA replication in two different ways (Supplementary Figure 7). DFdCMP is incorporated during primer extension by a DNA polymerase. The presence of dFdCMP at or near the 3′-end of the primer inhibits primer extension reaction, leaving an SSB. When the primer is extended fully [5, 6], the dFdCMP-containing strand is served as a template for the next round of DNA replication and dFdCMP in template blocks DNA chain elongation reaction [7]. Our results demonstrated that dFdCMP in the primer end can be removed by XPF-ERCC1 along with APE (Figures 1 and 2). In addition, our results showed that XPG-deficient UV135 cells are moderately sensitive to gemcitabine (Figure 1(b)). It has been also shown that nucleoside analogs are substrates for NER [28]. Thus, dFdCMP in the template will be removed by NER. It is noted that XPG plays an additional role outside of nucleotide excision repair. XPG interacts and stimulates a DNA glycosylase NTH1 and thus functions as a modulator of base excision repair [29–31]. NTH1 removes various oxidative DNA lesions including thymine glycol. Although there is no report of the NTH1 activity to dFdC in DNA, we cannot eliminate the possibility that XPG in concert with NTH1 removes dFdC in DNA. Thus, although our results in Figure 1(b) implicate that NER contributes to the repair of gemcitabine-induced DNA damage, additional experiments with other NER mutants such as XP-A cell lines are required to confirm this activity. DFdCMP in the template can also be bypassed by translesion DNA synthesis (TLS) by DNA polymerase eta (PolH) [7]. These three pathways could influence the cytotoxicity (thus the efficacy) of gemcitabine; therefore, the identification of genes/factors that impact these pathways is required for better evaluation of the potential efficacy of gemcitabine with an individual patient.

A MMR-deficiency has an adverse effect on gemcitabine. HCT116 that is MMR-deficient showed more resistance to gemcitabine compared to MMR-restored HCT116 [32]. The gemcitabine resistance in the MMR-deficient HCT116 might be caused by analogous mechanisms to MMR-dependent cytotoxicity of O^6^-methylguanine [33, 34], but a detailed mechanism is unclear. However, our results demonstrated that inactivation of XPF (or APE) could sensitize the MMR-deficient HCT116 cells to gemcitabine. The XPF- (and APE-) mediated repair pathway might be a potential target for the patients associated with a MMR-deficiency when gemcitabine is administered.

Our results and the reports from others found that BRCA2 (and likely other genes involved in HR) is required for the cytotoxicity of gemcitabine [24, 25]. BRCA2-deficient cells are more resistant to gemcitabine treatment compared to the BRCA2-restored cells (Figure 4). The mechanism of this phenomenon is not clear; however, DNA intermediate structures generated during BRCA2-mediated HR such as DNA polymerization step during HR might be stabilized and not properly processed in the presence of dFdCMP and/or dFdCTP. In the absence of HR, DSBs associated with gemcitabine might be repaired by a DSB repair pathway that is not involved in a long stretch of DNA synthesis such as alternative nonhomologous end-joining. Because there is a group of pancreatic cancer patients who are defective in HR [35, 36] and gemcitabine is used as a primary drug for the most of pancreatic cancer patients, the status of the HR activity in each patient should be carefully evaluated prior to treatment.

Our results showed that DNA repair activities greatly impact the efficacy of gemcitabine. Identification of DNA repair genes that influence the XPF- (and APE-) mediated repair pathway and an additional DNA repair pathway that modulates the cytotoxicity of gemcitabine is underway. In the era of personalized medicine, we should also develop a method to evaluate these DNA repair activities in patient samples to evaluate the potential efficacy of gemcitabine properly.

## Data Availability

The data supporting the findings of this study are available within the article and its supplementary information.

## Supplementary Materials

Supplementary Materials Supplementary Figure 1: the expression of the wild type human XPF and the endonuclease-deficient human XPF(DA) in CHO UV41 cells. Supplementary Figure 2: confirmation of DNA repair defective phenotypes in the XPF-suppressed cells. Supplementary Figure 3: basic characterizations of the XPF-defective HCT116 cells. Supplementary Figure 4: biochemical fractionations of cell lysates. Supplementary Figure 5: impact of gemcitabine on the recruitment of APE to chromatin. Supplementary Figure 6: characterizations of BRCA2-deficient cells and their HR-restored revertants. Supplementary Figure 7: proposed mechanism of the removal of gemcitabine-induced DNA lesions. Supplementary Table 1: list of surviving fractions with standard deviation of each experiment.

## References

[1] Bergman A., Peters G. (2006). Gemcitabine. Mechanism of action and resistance. *Cancer Drug Discovery and Development: Deoxynucleoside Analogs in Cancer Therapy*.

[2] Kroep J. R., Peters G. J., Nagourney R. A., Peters G. J. (2006). Clinical activity of gemcitabine as a single agent and in combination. *Cancer Drug Discovery and Development: Deoxynucleoside Analogs in Cancer Therapy*.

[3] De Sousa Cavalcante L., Monteiro G. (2014). Gemcitabine: metabolism and molecular mechanisms of action, sensitivity and chemoresistance in pancreatic cancer. *European Journal of Pharmacology*.

[4] Gandhi V., Legha J., Chen F., Hertel L. W., Plunkett W. (1996). Excision of 2',2'-difluorodeoxycytidine (gemcitabine) monophosphate residues from DNA. *Cancer Research*.

[5] Jiang H. Y., Hickey R. J., Abdel-Aziz W., Malkas L. H. (2000). Effects of gemcitabine and araC on in vitro DNA synthesis mediated by the human breast cell DNA synthesome. *Cancer Chemotherapy and Pharmacology*.

[6] Prakasha Gowda A. S., Polizzi J. M., Eckert K. A., Spratt T. E. (2010). Incorporation of gemcitabine and cytarabine into DNA by DNA polymerase *β* and ligase III/XRCC1. *Biochemistry*.

[7] Chen Y. W., Cleaver J. E., Hanaoka F., Chang C. F., Chou K. M. (2006). A novel role of DNA polymerase eta in modulating cellular sensitivity to chemotherapeutic agents. *Molecular Cancer Research*.

[8] Gregg S. Q., Robinson A. R., Niedernhofer L. J. (2011). Physiological consequences of defects in ERCC1-XPF DNA repair endonuclease. *DNA Repair*.

[9] Manandhar M., Boulware K. S., Wood R. D. (2015). The ERCC1 and ERCC4 (XPF) genes and gene products. *Gene*.

[10] McNeil E. M., Melton D. W. (2012). DNA repair endonuclease ERCC1-XPF as a novel therapeutic target to overcome chemoresistance in cancer therapy. *Nucleic Acids Research*.

[11] Abdullah U. B., McGouran J. F., Brolih S. (2017). RPA activates the XPF-ERCC1 endonuclease to initiate processing of DNA interstrand crosslinks. *EMBO Journal*.

[12] Fisher L. A., Bessho M., Bessho T. (2008). Processing of a psoralen DNA interstrand cross-link by XPF-ERCC1 complex in vitro. *The Journal of Biological Chemistry*.

[13] Klein Douwel D., Boonen R. A. C. M., Long D. T. (2014). XPF-ERCC1 acts in unhooking dna interstrand crosslinks in cooperation with FANCD2 and FANCP/SLX4. *Molecular Cell*.

[14] Klein Douwel D., Hoogenboom W. S., Boonen R. A. C. M., Knipscheer P. (2017). Recruitment and positioning determine the specific role of the XPF-ERCC1 endonuclease in interstrand crosslink repair. *EMBO Journal*.

[15] Wang A. T., Sengerová B., Cattell E. (2011). Human SNM1a and XPF-ERCC1 collaborate to initiate DNA interstrand cross-link repair. *Genes & Development*.

[16] Al-minawi A. Z., Saleh-gohari N., Helleday T. (2008). The ERCC1/XPF endonuclease is required for efficient single-strand annealing and gene conversion in mammalian cells. *Nucleic Acids Research*.

[17] Motycka T. A., Bessho T., Post S. M., Sung P., Tomkinson A. E. (2004). Physical and functional interaction between the XPF/ERCC1 endonuclease and hRad52. *The Journal of Biological Chemistry*.

[18] Bennardo N., Cheng A., Huang N., Stark J. M. (2008). Alternative-NHEJ is a mechanistically distinct pathway of mammalian chromosome break repair. *Plos Genetics*.

[19] Fisher L. A., Samson L., Bessho T. (2011). Removal of reactive oxygen species-induced 3′-blocked ends by XPF-ERCC1. *Chemical Research in Toxicology*.

[20] Zhang Y.-W., Regairaz M., Seiler J. A., Agama K. K., Doroshow J. H., Pommier Y. (2011). Poly(ADP-ribose) polymerase and XPF-ERCC1 participate in distinct pathways for the repair of topoisomerase I-induced DNA damage in mammalian cells. *Nucleic Acids Research*.

[21] Takahata C., Masuda Y., Takedachi A., Tanaka K., Iwai S., Kuraoka I. (2015). Repair synthesis step involving ERCC1-XPF participates in DNA repair of the Top1-DNA damage complex. *Carcinogenesis*.

[22] Caldecott K. W. (2008). Single-strand break repair and genetic disease. *Nature Reviews Genetics*.

[23] Lau J. P., Weatherdon K. L., Skalski V., Hedley D. W. (2004). Effects of gemcitabine on APE/ref-1 endonuclease activity in pancreatic cancer cells, and the therapeutic potential of antisense oligonucleotides. *British Journal of Cancer*.

[24] Im M. M., Flanagan S. A., Ackroyd J. J., Shewach D. S. (2015). Drug metabolism and homologous recombination repair in radiosensitization with gemcitabine. *Journal of Radiation Research*.

[25] Jones R. M., Kotsantis P., Stewart G. S., Groth P., Petermann E. (2014). BRCA2 and RAD51 promote double-strand break formation and cell death in response to gemcitabine. *Molecular Cancer Therapeutics*.

[26] Sakai W., Swisher E. M., Jacquemont C. (2009). Functional restoration of BRCA2 protein by secondary BRCA2 mutations in BRCA2-mutated ovarian carcinoma. *Cancer Research*.

[27] Sakai W., Swisher E. M., Karlan B. Y. (2008). Secondary mutations as a mechanism of cisplatin resistance in BRCA2-mutated cancers. *Nature*.

[28] Buschta-Hedayat N., Buterin T., Hess M. T., Missura M., Naegeli H. (1999). Recognition of nonhybridizing base pairs during nucleotide excision repair of DNA. *Proceedings of the National Academy of Sciences of the United States of America*.

[29] Bessho T. (1999). Nucleotide excision repair 3' endonuclease XPG stimulates the activity of base excision repair enzyme thymine glycol DNA glycosylase. *Nucleic Acids Research*.

[30] Klungland A., Höss M., Gunz D. (1999). Base excision repair of oxidative DNA damage activated by XPG protein. *Molecular Cell*.

[31] Scharer O. D. (2008). XPG: its products and biological roles. *Advances in Experimental Medicine and Biology*.

[32] Robinson B. W., Im M. M., Ljungman M., Praz F., Shewach D. S. (2003). Enhanced radiosensitization with gemcitabine in mismatch repair-deficient HCT116 cells. *Cancer Research*.

[33] Wang J. Y. J., Edelmann W. (2006). Mismatch repair proteins as sensors of alkylation DNA damage. *Cancer Cell*.

[34] York S. J., Modrich P. (2006). Mismatch repair-dependent iterative excision at irreparable O6-methylguanine lesions in human nuclear extracts. *The Journal of Biological Chemistry*.

[35] Rustgi A. K. (2014). Familial pancreatic cancer: Genetic advances. *Genes & Development*.

[36] Witkiewicz A. K., McMillan E. A., Balaji U. (2015). Whole-exome sequencing of pancreatic cancer defines genetic diversity and therapeutic targets. *Nature Communications*.

