# Supplementary material for: Inactivation of XPF Sensitizes Cancer Cells to Gemcitabine
Source: J Nucleic Acids. 2019 Mar 3;2019:6357609. doi: 10.1155/2019/6357609 (PMC6421022; doi:10.1155/2019/6357609)
Supplement: Supplementary Materials — Supplementary Figure 1: the expression of the wild type human XPF and the endonuclease-deficient human XPF(DA) in CHO UV41 cells. Supplementary Figure 2: confirmation of DNA repair defective phenotypes in the XPF-suppressed cells. Supplementary Figure 3: basic characterizations of the XPF-defective HCT116 cells. Supplementary Figure 4: biochemical fractionations of cell lysates. Supplementary Figure 5: impact of gemcitabine on the recruitment of APE to chromatin. Supplementary Figure 6: characterizations of BRCA2-deficient cells and their HR-restored revertants. Supplementary Figure 7: proposed mechanism of the removal of gemcitabine-induced DNA lesions. Supplementary Table 1: list of surviving fractions with standard deviation of each experiment. [file 6357609.f1.pdf]

Supplemental information

Supplemental Figure 1.

The expression of the wild type human XPF and the endonuclease deficient human XPF(DA) in CHO UV41 cells.

Supplemental Figure 2.

Confirmation of DNA repair defective phenotypes in the XPF-suppressed cells.

Supplemental Figure 3.

Basic characterizations of the XPF-defective HCT116 cells.

Supplemental Figure 4.

Biochemical fractionations of cell lysates.

Supplemental Figure 5.

Impact of gemcitabine on the recruitment of APE to chromatin.

Supplemental Figure 6.

Characterizations of BRCA2-deficient cells and their HR-restored revertants.

Supplemental Figure 7.

Proposed mechanism of the removal of gemcitabine-induced DNA lesions.

Supplemental Table 1.

List of surviving fraction with standard deviation of each experiment.

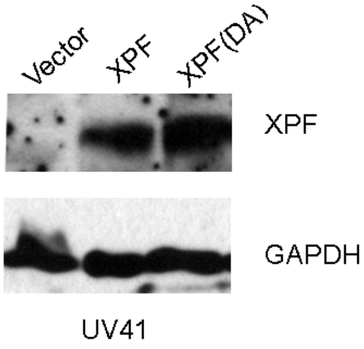

Supplemental Figure 1. The expression of human XPF in the XPF-defective UV41 cells. The human XPF gene was expressed in Chinese Hamster Ovary cell line, UV41. Cell lysates (100 µg) from UV41 cells with a control vector (Vector), wild type human XPF (XPF) or the endonuclease defective XPF(DA), were analyzed by the western blotting. Wild type human XPF and the endonuclease defective XPF(DA) were expressed at a similar level. GADPH was used as loading control.

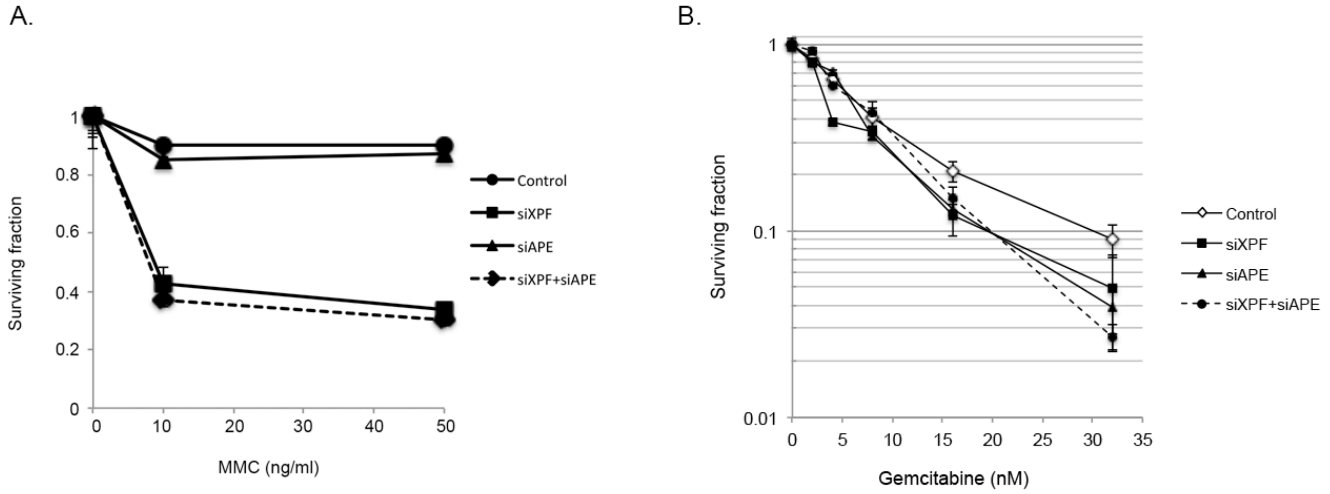

Supplemental Figure 2. Confirmation of a DNA repair defective phenotype in the XPF-suppressed cells. (A) MMC-sensitivity of the XPF-suppressed HeLa cells was examined. HeLa cells treated with siXPF alone, siAPE alone, or the combination of siXPF and siAPE were treated with the indicated concentration of mitomycin C (MMC). Cells with siXPF alone and the combination of siXPF and siAPE showed sensitivity to MMC ($p<0.01$), while siAPE-treatment alone did not result in the MMC-sensitivity. Three independent experiments were performed and averages of surviving fraction are plotted. The error bars show standard deviations.
(B) Sensitization of HeLa cells to gemcitabine by the suppression of XPF or APE. HeLa cells treated with siXPF alone, siAPE alone or the combination of siXPF and siAPE were treated with the indicated concentration of gemcitabine. Cells with siXPF alone and the siAPE alone showed sensitivity to gemcitabine at 32 nM ($p<0.05$). Surviving fractions at 32 nM of gemcitabine were 0.090±0.018 for siControl, 0.049±0.026 for siXPF and 0.039±0.011 for siAPE. A simultaneous suppression of XPF and APE-treatment did not change the gemcitabine-sensitivity. Three independent experiments were performed and averages of surviving fraction are plotted. The error bars show standard deviations.

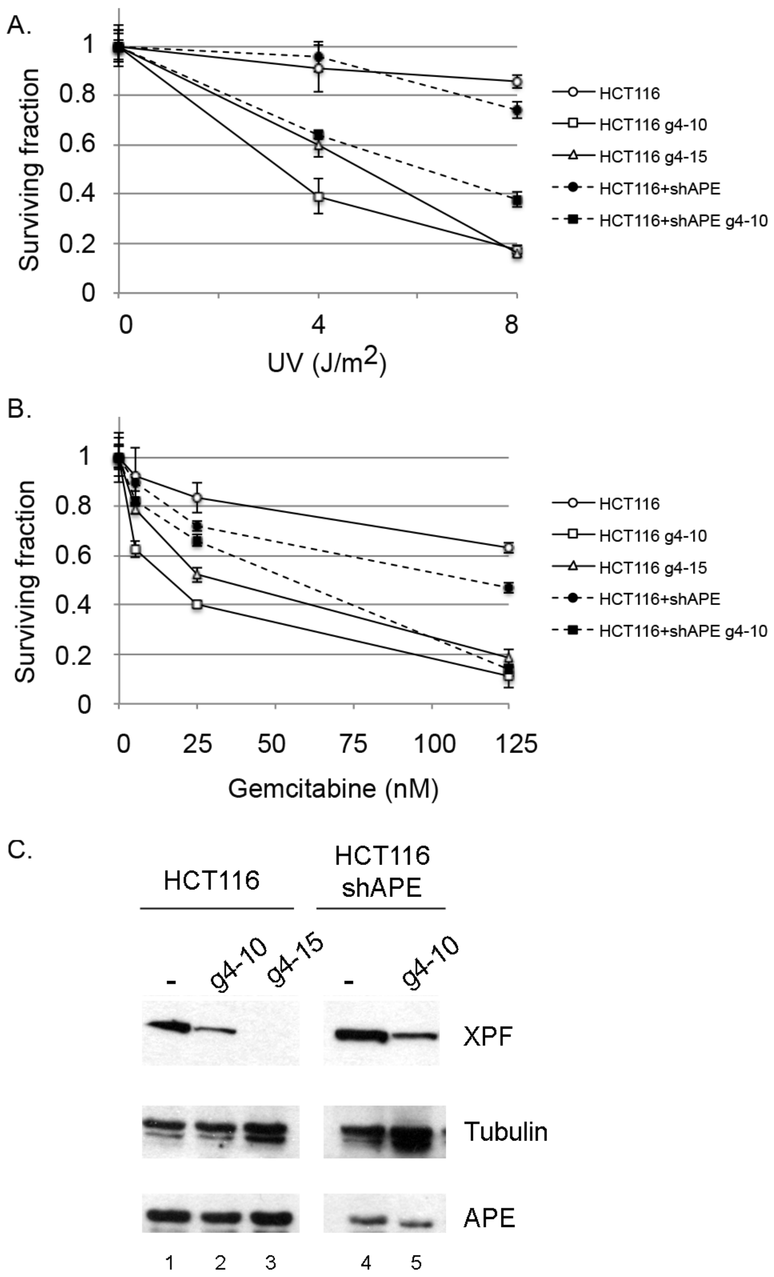

Supplemental Figure 3. Characterizations of the XPF-defective HCT116 cells.
XPF-deficient HCT116 cells were generated by CRISPR/Cas9 technology. (A, B) The XPF-deficient cells showed the UV-sensitive phenotype due to a defect in nucleotide excision repair and also displayed the sensitivity to gemcitabine. The XPF mutant with the APE suppression (HCT116 shAPE g4-10) showed similar sensitivity to gemcitabine to the XPF-mutants (HCT116 g4-10 and g4-15), confirming the epistatic relationship between the XPF- deficiency and the APE-deficiency in the gemcitabine-induced cytotoxicity. Three independent experiments were performed and averages of surviving fraction are plotted. The error bars show standard deviations. Surviving fractions, standard deviations and p-values are listed in Supplemental Table 1. (C) Western blots showed significant reductions of the expression of XPF in HCT116 g4-10 (~80% reduction in lane 2) and g4-15 (>95% reduction in lane 3), and HCT116 shAPE g4-10 (~90% reduction in lane 5). APE was suppressed by ~50% in HCT116 shAPE cell lines (compare lanes 1 and 4). Tubulin was used as loading control for the western blots. Five µg of cell lysate from each cell line was analyzed.

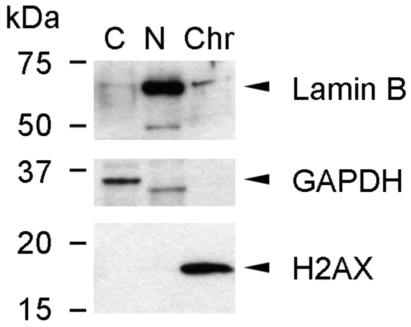

Supplemental Figure 4. Biochemical fractionations of cell lysates. Western blots demonstrate typical results of the fractionations of cytosolic (C), nuclear (N), and chromatin (Chr) fractions. GAPDH, lamin B and Histone H2AX were used as markers for cytosol, nuclear and chromatin.

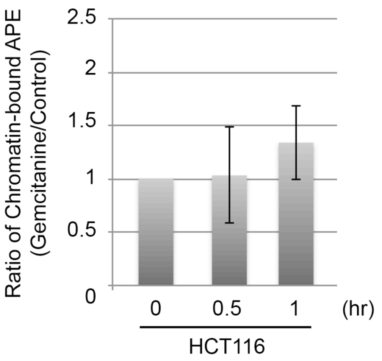

Supplemental Figure 5. The gemcitabine-treatment does not change the amount of APE on chromatin. Levels of the chromatin-bound APE were not changed after the treatment with 1 μM gemcitabine. The averages from three independent experiments were depicted as a bar graph. The error bars represent standard deviations from three independent experiments.

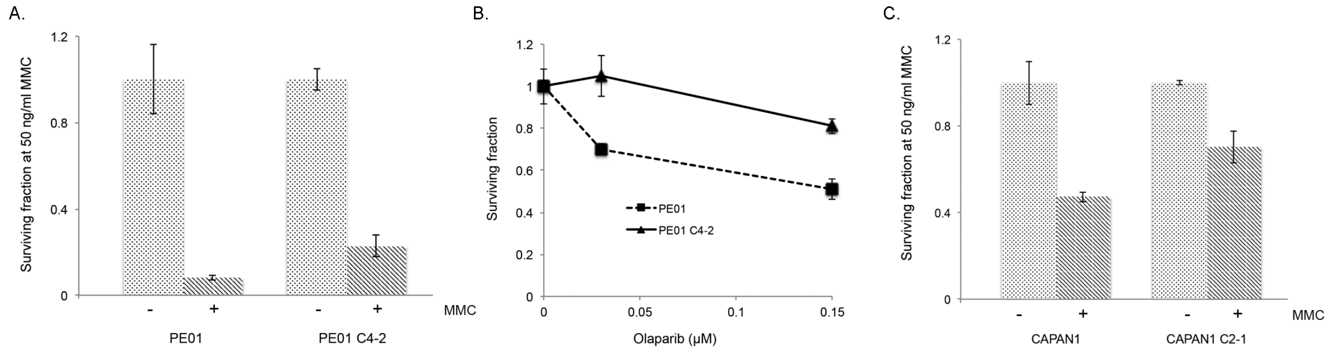

Supplemental Figure 6. Characterizations of BRCA2-deficient cells and their revertants. (A, C) BRCA2-deficient PE01 and CAPAN1 showed the MMC-sensitivity and their BRCA2-revertants, PE01(C4-2) and CAPAN1(C2-1) restored the MMC-resistance (with $p<0.01$). Cells were treated with 50 ng/ml MMC for two hours. After removing MMC, the cells were grown in fresh medium for 5-7 days. (B) PE01 also displayed sensitivity to PARP-inhibitor, olaparib, while PE01(C2-1) was resistant to olaparib. These data confirm that PE01 and CAPAN1 are defective in BRCA2-mediated homologous recombination and their BRCA2-revertants regain the HR activity. Three independent experiments were performed and averages of surviving fraction are plotted. The error bars show standard deviations.

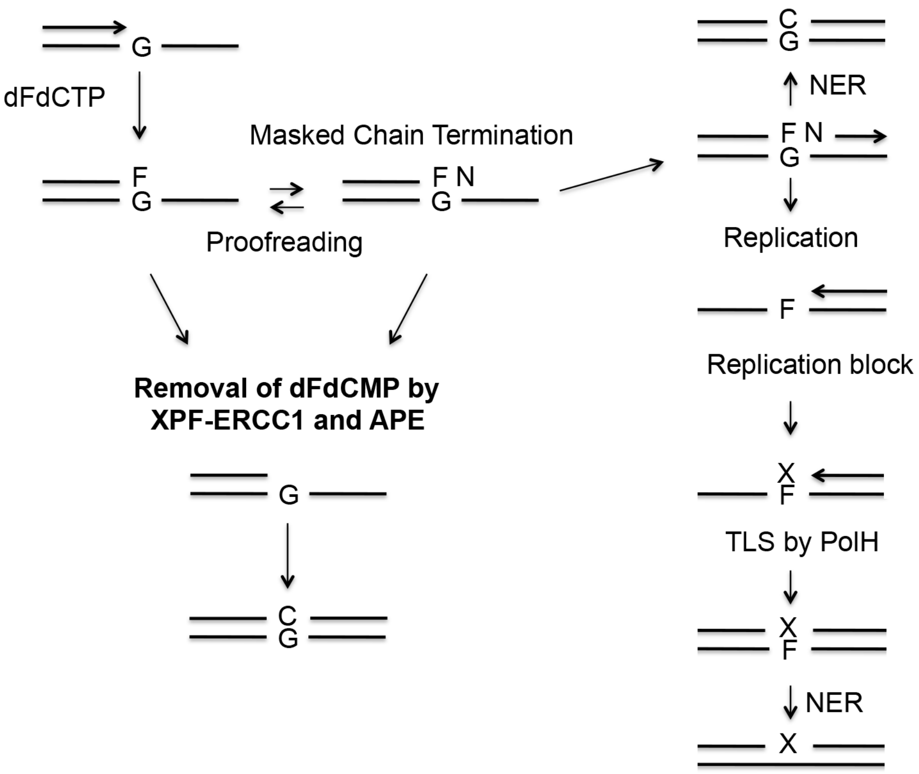

Supplemental Figure 7. Proposed mechanism of the removal of gemcitabine-induced DNA lesions. Please see the Discussion for the detail.

Supplemental Table 1
Surviving fraction and standard deviation at each concentration of gemcitabine

Figure 1: UV41

Cell line/treatment	Gemcitabine (nM)	Surviving Fraction	Standard Deviation
Vector	0	1	0.010
	0.1	0.976	0.056
	0.4	0.843	0.048
	1.6	0.639	0.032
	6.4	0.163	0.036
XPF	0	1	0.016
	0.1	1.01	0.063
	0.4	0.967	0.040
	1.6	0.869	0.021
	6.4	0.520	0.021
XPF(DA)	0	1	0.023
	0.1	0.943	0.005
	0.4	0.761	0.031
	1.6	0.681	0.023
	6.4	0.179	0.041

Figure 2 : HeLa siXPF/siAPE

Cell line/treatment	Gemcitabine (nM)	Surviving Fraction	Standard Deviation	*P-value
siControl	0	1	0.112	
	2	0.965	0.070	
	10	0.453	0.069	
	50	0.081	0.010	
siXPF	0	1	0.073	
	2	0.746	0.089	0.032
	10	0.174	0.055	0.0025
	50	0.018	0.020	0.00027
siAPE	0	1	0.050	
	2	0.762	0.105	0.060
	10	0.205	0.055	0.0015
	50	0.032	0.011	0.011
siXPF+siAPE	0	1	0.060	
	2	0.718	0.088	0.057
	10	0.187	0.049	0.0078
	50	0.037	0.020	0.0011

*Comparison of siXPF, siAPE or siXPF+siAPE with Control at each concentration of gemcitabine.

Supplemental Figure 2B: HeLa siXPF/siAPE

Cell line/treatment	Gemcitabine (nM)	Surviving Fraction	Standard Deviation	*P-value
siControl	0	1	0.020	
	2	0.849	0.082	
	4	0.656	0.055	
	8	0.415	0.047	
	16	0.210	0.027	
	32	0.090	0.018	
siXPF	0	1	0.020	
	2	0.819	0.030	0.501
	4	0.389	0.010	0.008
	8	0.343	0.010	0.076
	16	0.127	0.026	0.030
	32	0.049	0.026	0.043
siAPE	0	1	0.020	
	2	0.819	0.030	0.501
	4	0.715	0.017	0.121
	8	0.322	0.012	0.027
	16	0.133	0.010	0.015
	32	0.039	0.011	0.006
siXPF+siAPE	0	1	0.076	
	2	0.926	0.038	0.297
	4	0.603	0	0.437
	8	0.439	0.065	0.027
	16	0.158	0.021	0.011
	32	0.027	0.004	0.016

*Comparison of siXPF, siAPE or siXPF+siAPE with Control at each concentration of gemcitabine.

HCT116 cell lines

Supplemental Figure 3A: UV

Cell line/treatment	UV (J/m2)	Surviving Fraction	Standard Deviation	*P-value
HCT116	0	1	0.081	
	4	0.910	0.096	
	8	0.857	0.026	
HCT116 g4-10	0	1	0.064	
	4	0.391	0.071	0.0057
	8	0.175	0.017	0.0019
HCT116 g4-15	0	1	0.024	
	4	0.601	0.050	0.0025
	8	0.162	0.014	0.0021
HCT116 shAPE	0	1	0.054	
	4	0.956	0.062	0.393
	8	0.741	0.032	0.032
HCT116 shAPE g4-10	0	1	0.065	
	4	0.642	0.018	0.015
	8	0.378	0.031	0.0055

Supplemental Figure 3B:gemcitabine

Cell line/treatment	Gemcitabine (nM)	Surviving Fraction	Standard Deviation	*P-value
HCT116	0	1	0.101	
	5	0.924	0.112	
	25	0.834	0.059	
	125	0.631	0.022	
HCT116 g4-10	0	1	0.076	
	5	0.627	0.033	0.0046
	25	0.401	0.012	0.0020
	125	0.109	0.043	0.0071
HCT116 g4-15	0	1	0.049	
	5	0.739	0.018	0.031
	25	0.497	0.028	0.0081
	125	0.333	0.037	0.0075
HCT116 shAPE	0	1	0.043	
	5	0.895	0.034	0.196
	25	0.720	0.019	0.018
	125	0.469	0.054	0.020
HCT116 shAPE g4-10	0	1	0.041	
	5	0.820	0.018	0.060
	25	0.661	0.024	0.014
	125	0.137	0.024	0.0048

*Comparison of each mutant cell line to HCT116 at each UV dose and concentration of gemcitabine.
